# Early ERPs dissociate subjectively nonconscious low- and high-level face processing

**DOI:** 10.1093/nc/niaf025

**Published:** 2025-08-19

**Authors:** Maximilian Bruchmann, Josephine Skutta, Sebastian Schindler, Insa Schlossmacher, Torge Dellert, Thomas Straube

**Affiliations:** Institute of Medical Psychology and Systems Neuroscience, University of Münster, Von-Esmarch-Street 52, D-48149 Münster, Germany; Otto Creutzfeldt Center for Cognitive and Behavioral Neuroscience, University of Münster, Fliednerstr. 21, D-48149 Münster, Germany; Institute of Medical Psychology and Systems Neuroscience, University of Münster, Von-Esmarch-Street 52, D-48149 Münster, Germany; Institute of Medical Psychology and Systems Neuroscience, University of Münster, Von-Esmarch-Street 52, D-48149 Münster, Germany; Otto Creutzfeldt Center for Cognitive and Behavioral Neuroscience, University of Münster, Fliednerstr. 21, D-48149 Münster, Germany; Institute of Medical Psychology and Systems Neuroscience, University of Münster, Von-Esmarch-Street 52, D-48149 Münster, Germany; Otto Creutzfeldt Center for Cognitive and Behavioral Neuroscience, University of Münster, Fliednerstr. 21, D-48149 Münster, Germany; Institute of Medical Psychology and Systems Neuroscience, University of Münster, Von-Esmarch-Street 52, D-48149 Münster, Germany; Otto Creutzfeldt Center for Cognitive and Behavioral Neuroscience, University of Münster, Fliednerstr. 21, D-48149 Münster, Germany; Institute of Medical Psychology and Systems Neuroscience, University of Münster, Von-Esmarch-Street 52, D-48149 Münster, Germany

**Keywords:** EEG/ERP, subliminal, face, scramble, nonconscious processing, perceptual awareness scale

## Abstract

There is an ongoing debate about the extent to which faces are processed if they are not consciously perceived. In the present study, we used event-related potentials (ERPs) to investigate neural responses to faces and two types of control stimuli (monochrome color-matched ovals and Fourier phase-scrambled faces), which allowed us to dissociate low-level and high-level face processing. Based on a pre-registered sequential Bayesian sampling protocol, we recorded the electroencephalogram (EEG) from 40 participants and compared the average amplitude of early components of the ERP (P1, N170) between faces, scrambles, and blanks presented for 17 ms, while the mask followed directly or 200 ms after the target stimulus. Participants were asked to rate their subjective perception after each trial on a perceptual awareness scale, and only trials with the lowest rating in the masked condition were considered as subjectively nonconscious. Matching the pre-registered hypotheses, P1 amplitudes were higher for faces and scrambles compared to blanks but did not differ between faces and scrambles. This pattern was found for conscious and nonconscious faces, however, with smaller yet reliable differences in the latter case. In contrast, the N170 reliably differentiated between faces and both types of control stimuli (scrambles and blanks), again for conscious and, with attenuated differences, also for nonconscious faces. Findings support the hypothesis of two early stages of face processing, which are at least partially independent of consciousness awareness of stimuli. The P1 stage is associated with low-level processing, while the N170 reflects processing of face-related configural information.

## Introduction

Faces are considered a special category of visual stimuli in the way that their social and behavioral importance created an evolutionary pressure resulting in highly efficient and specialized neural processing. Although faces are complex and multidimensional stimuli, they are rapidly detected and recognized (Crouzet et al. 2010). Face processing is associated with specific neuronal networks (see Duchaine and Yovel 2015) and electrophysiological indices (Bentin et al. 1996; Eimer 2000).

There is an ongoing debate about the extent to which face processing occurs even in the absence of conscious perception of the faces. In this context, nonconscious face processing often refers to the modulation of neural activity by different properties of faces, such as their identity, emotional expression, orientation, or configural features that distinguish faces from control stimuli. At present, there appears to be no clear evidence for or against nonconscious processing of emotional expressions (e.g. Jiang et al. 2009; Yang et al. 2007; Bruchmann et al. 2025; but see Schlossmacher et al. 2017, 2025; for reviews, see e.g. Lanfranco et al. 2023; Mudrik and Deouell 2022), or face identity (for reviews, see Axelrod et al. 2015; Mudrik and Deouell 2022). In contrast, nonconscious processing of face configuration has been shown in several studies (Jiang et al. 2007; Suzuki and Noguchi 2013; Stein and Peelen 2021). Nevertheless, some studies do not support this conclusion (Harris et al. 2011; Rodríguez et al. 2012; Shafto and Pitts 2015; Dellert et al. 2021; Lanfranco et al. 2024). In particular, electrophysiological studies yield inconsistent outcomes, as reviewed in the following paragraphs.

Electrophysiological studies enable the investigation of the time course of face processing. The earliest component of the event-related potential (ERP) that reliably differentiates between visible facial and non-facial stimuli is the N170 (e.g. Bentin et al. 1996; Itier and Taylor 2004; Rousselet et al. 2004). The N170 is considered a structural encoding component and reliably found to be enlarged for faces compared to objects (Eimer 2000) or Fourier phase-scrambled faces (Rossion and Caharel 2011). The P1, which occurs about 100 ms after stimulus onset, has also been associated with face processing in some studies (Thierry et al. 2007; Dering et al. 2011; Wang et al. 2015), but seems to be strongly driven by low-level visual differences between stimuli (Bruchmann et al. 2020; Schindler et al. 2021). Correspondingly, two studies have shown that the P1 does not differ between phase-scrambled and real faces (Rossion and Caharel 2011; Civile et al. 2018).

Regarding the processing of nonconscious faces, findings differ strongly between studies. A magnetoencephalographic study presenting faces and houses under continuous flash suppression (CFS) found reduced but significant M170 enhancements for faces compared to houses (Sterzer et al. 2009). An ERP-CFS study reported an increased N170 for consciously perceived inverted compared to upright faces but a reversed effect in the nonconscious condition (Suzuki and Noguchi 2013). However, two studies using inattentional blindness to prevent conscious perception found no significant N170 effects between schematic faces and random line patterns (Shafto and Pitts 2015; Dellert et al. 2021). Furthermore, a backward masking study also observed no significant N170 differences; however, the data indicated a difference that might have been overlooked due to low statistical power (*N* = 11; Rodríguez et al. 2012). A very recent study with very brief subliminal stimulus presentation found no P1 and N170 effect to nonconscious faces (Lanfranco et al. 2024). Also, Harris et al. (2011), who used a sandwich masking paradigm, found no N170 differences between nonconsciously processed faces and houses. However, both N170 and P1 showed increased amplitudes across face and house trials compared to blank trials. In a recent high-powered backward masking study (*N* = 64; Bruchmann et al. 2025), we also observed both P1 and N170 differences between nonconscious faces and blank stimuli. The P1 differences between masked faces and blanks, however, were only discovered *via* an exploratory analysis outside the preregistered interval of interest. This effect, therefore, needs to be replicated. Furthermore, it remains unclear whether this effect is driven by low-level or configural features of faces. To differentiate between these possibilities, suited control stimuli, such as phase-scrambled faces, would be necessary.

Taken together, it appears that the likelihood of detecting early ERP effects for unseen faces compared to control stimuli increases with the statistical power of a given study, based on stimulus strengths, trial number, and sample size (see also Bruchmann et al. 2025). Furthermore, based on studies with consciously perceived stimuli (Rossion and Caharel 2011; Schindler et al. 2021), it seems likely that the functions of the P1 and N170 differ also for unconsciously processed stimuli. P1 effects on stimuli that are not consciously perceived could be mainly driven by low-level properties. In contrast, the N170 stage should reflect unconscious configural processing, at least when comparing faces with scrambles. However, both the occurrence of effects and their dependency on stimulus features have to be investigated in suitable studies in order to answer the question of to what extent face information can be processed unconsciously.

With the present preregistered backward masking study (https://osf.io/tajn9/), we aimed to answer this open question. First, we sought to replicate the findings of Bruchmann et al. (2025) regarding nonconscious differentiation between faces and blanks and test whether these effects reflected low-level stimulus differentiation or nonconscious face processing. To this end, we compared oval-cutout faces with monochrome color-matched ovals as in our previous study (Bruchmann et al. 2025), but additionally included oval-cutout scrambled faces, constructed *via* a novel approach that matches the Fourier spectrum within the oval target region to that of the faces (see Supplement). These scrambles can, therefore, serve as an optimal control stimulus for the low-level properties of the face stimuli. Second, an empirical problem in several studies pertains to the way consciousness is controlled and measured (Sterzer et al. 2014; Kerr et al. 2017; Moors 2019; Moors et al. 2019; Lanfranco et al. 2023; Prieto et al. 2025). The current study assessed awareness on a trial-by-trial basis using a suited Perceptual Awareness Scale (PAS) offering sufficiently sensitive behavioral measures (Overgaard et al. 2010; Sandberg and Overgaard 2015). Third, the present study used a sufficiently large sample (determined *via* Bayesian sampling stopping criteria) to address the question of sufficient sample sizes.

We hypothesized that P1 amplitudes would be identical for faces and scrambles but lower for blanks. This difference between stimuli with and without low-level contrast modulations was expected in the conscious and, to a reduced degree, also in the nonconscious condition. For the N170, we hypothesized that faces produce larger amplitudes than scrambles, again both for the conscious and, to a reduced degree, the nonconscious condition.

## Materials and methods

### Participants

We recruited a total of 45 participants at the University of Münster. All participants had normal or corrected-to-normal vision, were right-handed, and had no reported history of neurological or psychiatric disorders. Two participants were excluded due to poor EEG data (i.e. fewer than 40 trials per condition remained after removing trials with artifacts; see EEG recording and preprocessing), and three participants were excluded because their response bias did not meet the predefined criterion (see Results section). This resulted in a final sample of 40 participants (30 female, 10 male, and 0 diverse) with a mean age of 23.58 years (SD = 2.35, range = 20–32). Participants gave written informed consent and were paid or received course credit for their participation. We tested for nonconscious face-scramble differences using a Bayesian t-test (Keysers et al. 2020). As registered, we tested a minimum of 40 healthy participants but stopped sampling when the Bayes factor (BF) showed at least moderate evidence in favor of a larger N170 for nonconscious faces compared to scrambles or in favor of a null effect (BF_10_ > 3 or BF_10_ < 1/3). The stopping rule was fulfilled after the above-described sample of 40 participants was recorded, and thus, no further measurements were performed.

### Apparatus and stimuli

For target faces, we used 40 female and 40 male individuals who displayed neutral expressions. The target faces' ages ranged approximately between 18 and 40 years. The stimuli were taken from different face sets: the FACES database (Ebner et al. 2010), the NimStim set (Tottenham et al. 2009), and the Radboud Faces Database (Langner et al. 2010). All images were restricted to an oval cutout, which removes the ears and most of the hair. The transition between the oval center and the background was blurred with a Gaussian filter. Masks were created by cutting the oval face region of each image into squares of 10 × 10 pixels and rearranging these squares randomly. The same oval cutout and edge blur were applied to these images. This procedure resulted in 80 masks, each corresponding to one face image. Two types of control stimuli were used, referred to as blanks and scrambles. Blanks consisted of a homogenously colored oval with blurred edges, the color corresponding to the average RGB color of the oval face region of each individual face. Scrambles were created by a custom algorithm producing control stimuli whose Fourier spectrum within the oval target region was matched to the spectrum of the faces (see Supplementary Material for details). Thus, blanks matched faces in average color and luminance but contained no contrast modulations within the target region. Scrambles matched faces in average color and contrast, and additionally in contrast as defined by their Fourier power spectrum.

The total set consisted of 80 faces, 80 matched blanks, and 80 matched scrambles. An additional set of four identities not used for the stimuli described above was used for practice trials.

### Procedure

**Figure 1:**
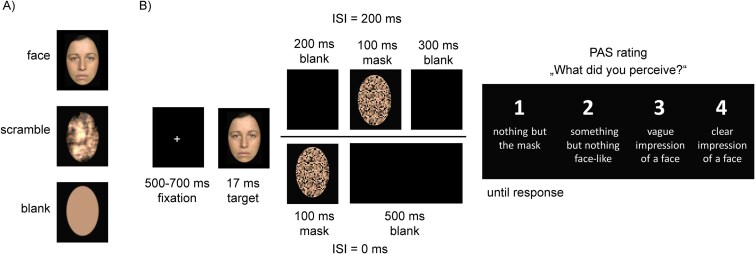
Trial and stimulus illustration. (A) Examples of each stimulus type. (B) Temporal stimulus sequence. The face example shown here was taken from the Radboud Face Database. Please note that the authors of the Radboud Face Database allow presenting images from the database in purely scientific publications (https://rafd.socsci.ru.nl/RaFD2/RaFD?p=faq).

The experiment was programmed and run using Matlab (version R2022a; MathWorks Inc., Natick, MA; http://www.mathworks.com) and the Psychophysics Toolbox (http://psychtoolbox.org/; Version 3.0.19; Brainard 1997; Kleiner et al. 2007). Participants were instructed to avoid eye movements and blinks during the stimulus presentation. They were prepared for the EEG while filling out a demographic questionnaire. The main experiment consisted of a backward masking design. Each trial began with a fixation cross in the center of the screen for a randomized duration between 500 and 700 ms. Next, the target stimulus (face, scramble, or blank) was presented for 17 ms. In order to vary the degree of conscious visibility, we manipulated the ISI between the target face and the mask: in the ISI0 condition, the masks' onset coincided with the target offset, whereas in the ISI200 condition, the target offset and mask onset were separated by an empty screen lasting for 200 ms (see Fig. 1). We expected most target faces to remain nonconscious in the ISI0 condition and to be conscious in the ISI200 condition. Subjective awareness was assessed by prompting the participants 600 ms after the offset of the target stimulus to rate their perception on an adjusted version of the PAS (Overgaard et al. 2010; Sandberg and Overgaard 2015; Overgaard and Sandberg 2021). Participants were asked whether they had not noticed anything (“nothing but the mask”; PAS = 1), had seen “something but nothing face-like” (PAS = 2), had a “vague impression of a face” (PAS = 3), or had a “clear impression of a face” (PAS = 4). After each key press, the selected response was surrounded by a white frame presented for 200 ms. Within each ISI condition, each target stimulus was presented twice. This resulted in 960 trials. Four different pseudorandomized trial sequences were generated by shuffling the order of trials until no condition (defined by stimulus type and ISI) was repeated more than 3 times consecutively. The four trial sequence versions were balanced across participants. Before the main experiment, participants performed 24 practice trials to get accustomed to the stimulus presentation and the response keys.

### Behavioral data analysis

Behavioral data were analyzed using Matlab (version R2022a; MathWorks Inc., Natick, MA; http://www.mathworks.com) and JASP (version 0.11.1; JASP Team 2016). The analysis was based on each PAS level's relative frequency and measures for detection sensitivity (*d*′) and response criterion (*c*) based on signal detection theory (SDT; Green and Swets 1966). SDT measures were based on a dichotomization of the PAS ratings as follows: for the ISI0 condition, PAS = 1 ratings counted as a “no” response, and PAS > 1 as a “yes” response. For the ISI200 condition, PAS ≤ 2 was counted as “no” responses, and PAS ≥ 3 responses were counted as “yes” responses. This asymmetric approach was chosen because, for ISI0, we aimed to obtain a maximally conservative definition of (the absence of) awareness, i.e. *d*' = 0 should index the inability to detect anything. Therefore, the detection of anything more than the mask stimulus counted as a hit. For the ISI200 condition, the measure reflected the conscious discrimination between both control stimuli and faces. Therefore, only perceived face features counted as a hit. We acknowledge that, optimally, yes-no-responses and subjective ratings should be obtained separately, which would also allow for a dissociation of objective performance measures (*d*′) from metacognitive ratings (meta-*d*′; Fleming 2017; Maniscalco and Lau 2012, 2014). We opted for a single PAS rating per trial to maximize the number of trials per subject and because the EEG analysis relies on trials that were rated as either subjectively conscious (PAS > 1) or nonconscious (PAS = 1), taking advantage of the optimal sensitivity of the PAS scale (Sandberg and Overgaard 2015; however, see Fahrenfort et al. 2025 for a recent critique of this approach). The SDT measures for sensitivity (*d*′) and the response criterion (*c*; Hautus et al. 2021) obtained from a dichotomization of PAS ratings are thus potentially distorted by metacognitive processes and may violate assumptions of SDT. We nevertheless used these measures to exclude participants with extreme response criteria and performed 1-sample t-tests of *d*′ and *c* in the ISI0 and ISI200 conditions as pre-registered.

Response rates of 0% and 100% were replaced by 0.3125% and 99.6875%, corresponding to 0.5 out of 160 trials (Macmillan and Kaplan 1985). To identify and exclude participants with extremely conservative or liberal response criteria, we calculated c per participant, averaged across conditions, and then z-standardized across participants. All further analyses excluded participants with |*z*_c_| > 2.5, as these could lead to an under- or overestimation of nonconscious processing. Since for ISI0 and ISI200 the measures *d*′ and *c* were calculated differently, we chose not to compare them directly and thus did not specify any hypotheses. *P*-values are only reported as significant if they passed the respective false discovery rate threshold (FDR; Benjamini and Hochberg 1995) that controls for multiple comparisons.

### E‌EG recording and preprocessing

EEG signals were recorded from 64 BioSemi active electrodes using Biosemi's ActiView software (www.biosemi.com). Four additional electrodes measured horizontal and vertical eye movement. The recording sampling rate was 512 Hz. Offline, the data were re-referenced to average reference and band-pass filtered from 0.01 (6 dB/octave) to 40 (24 dB/octave) Hz. Recorded eye movements were corrected using BESA's automatic eye-artifact correction method (Ille et al. 2002). Filtered data were segmented from 200 ms before stimulus onset until 800 ms after stimulus presentation. Trials amplitudes were declared as out of range based on a semi-automatic procedure implemented in BESA which defines an artifact threshold based on absolute amplitudes (>120 μV), the signal gradient (75 μV/sample) and low signal (<0.01 μV). This threshold was adjusted individually based on eye-inspection of the overall data quality. Bad electrodes (based on visual inspection) were interpolated using a spline interpolation procedure (*M* = 3.39, SD = 2.97, Min = 0, Max = 12). Baseline correction was used at the 200 ms interval before stimulus onset.

The dependent variable for all ERP analyses was mean amplitudes averaged over the following time windows and channels: We preregistered to examine the mean amplitude for the P1 from 80 to 100 ms and for the N170 from 130 to 170 ms. The preregistered sensors of interest for both components consisted of two symmetrical occipital clusters (left P9, P7, PO7; right P10, P8, PO8). ERPs were calculated by averaging conscious and nonconscious trials separately for each target stimulus type (face, scramble, blank). Nonconscious trials were defined as trials with ISI = 0 and PAS = 1. Conscious trials were defined as trials with ISI = 200 ms and PAS > 1.

### Statistical analyses and hypotheses

ERPs were statistically analyzed using a three (stimulus type: face, scramble, and blank) by two (consciousness: conscious vs. nonconscious) repeated-measures analyses of variance (ANOVA) per component (P1 and N170). The resulting *P*-values of the six F-tests are only reported as significant if they passed the respective FDR threshold (Benjamini and Hochberg 1995).

Partial eta-squared (η_P_^2^) was used to describe effect sizes (Cohen 2013). For the P1, we expected a main effect of consciousness due to the different temporal overlaps of ERPs generated by the mask and the target. We further expected a main effect of stimulus type, due to higher P1 amplitudes to stimuli with contrast modulation (faces and scrambles) than without (blanks), and an interaction of stimulus type and consciousness due to larger stimulus type effects in the conscious compared to the nonconscious condition. Crucially, we preregistered the following planned comparisons to be tested using Bayesian t-tests: for conscious as well as nonconscious trials, we expected increased P1 amplitudes for faces compared to blanks and for scrambles compared to blanks. For the comparison of faces and scrambles, we expected an absence of differences, for conscious as well as nonconscious trials, due to their matched low-level properties. Face-blank and scramble-blank differences were expected to be larger in the conscious compared to the nonconscious condition.

For the N170, we again expected a main effect of consciousness due to the different temporal overlaps, a main effect of stimulus type, due to higher N170 amplitudes (please note that in case of the negative-going N170, “higher” will always refer to “more negative”) to faces compared to non-faces (scrambles and blanks), and an interaction of stimulus type and consciousness due to larger stimulus type effects in the conscious compared to the nonconscious condition. For the N170, we preregistered the following planned comparisons: for conscious as well as nonconscious trials, we expect increased N170 amplitudes for faces compared to scrambles and for faces compared to blanks. We expected the face-scramble difference to be larger for conscious compared to nonconscious trials.

BFs were labeled using Lee and Wagenmakers' (2014) scale. BF_10_ denotes Bayes factors favoring the alternative hypothesis, and BF_01_ denotes Bayes factors favoring the null hypothesis. One-sided Bayesian t-tests were chosen whenever hypotheses included the direction of an effect. Two-sided Bayesian t-tests were chosen whenever the null hypothesis was predicted to be true.

## Results

### Behavior

#### Sensitivity and bias

**Figure 2:**
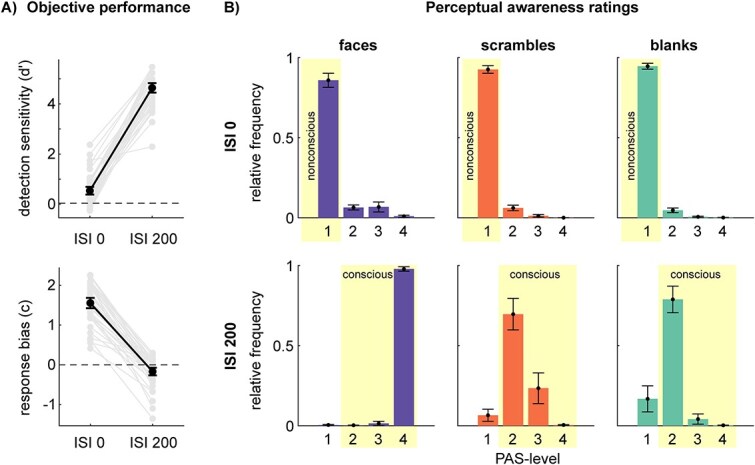
Behavioral data for faces, scrambles, and blanks. (A) Objective performance measures for detection sensitivity (*d*′; top plot) and response bias (*c*; bottom plot; positive values indicate a conservative bias). Dots represent individual participants (*N* = 40, i.e. after application of the exclusion criteria). (B) Distribution of perceptual awareness ratings for faces (left column of bar plots), scrambles (middle column of bar plots), and blanks (right column of bar plots), presented with a target-mask ISI of 0 ms (top row) or 200 ms (bottom row). The yellow rectangles highlight the conditions labeled as nonconscious and conscious, respectively, for the EEG analyses. All error bars represent 95% confidence intervals of the mean.

Figure 2A depicts the objective measures of performance (*d*′ and *c*). One-sample t-tests revealed that *d*′ differed from 0 significantly for ISI0 (*t*_(39)_ = 5.655, *P* < .001, Cohen’s *d* = 0.894) and for ISI200 (*t*_(39)_ = 39.704, *P* < .001, Cohen’s *d* = 6.278). Also, c differed significantly from 0 for ISI0 (*t*_(39)_ = 19.401, *P* < .001, Cohen’s d = 3.068) and for ISI200 (*t*_(39)_ = −2.861, *P* = .014, Cohen’s *d* = −0.452). As noted above, *d*′ and *c* cannot be directly compared between the ISI conditions but are included to illustrate individual and average performance.

#### Exclusion due to extreme response biases

Three participants were excluded due to an extreme response bias, defined as |*z*_c_| > 2.5, leading to the preregistered sample size of *N* = 40.

#### PAS ratings

Figure 2B shows the distribution of PAS ratings to illustrate which conditions were labeled as nonconscious or conscious for the EEG analyses. We did not perform any statistical analyses of these distributions.

#### ERPs

**Figure 3:**
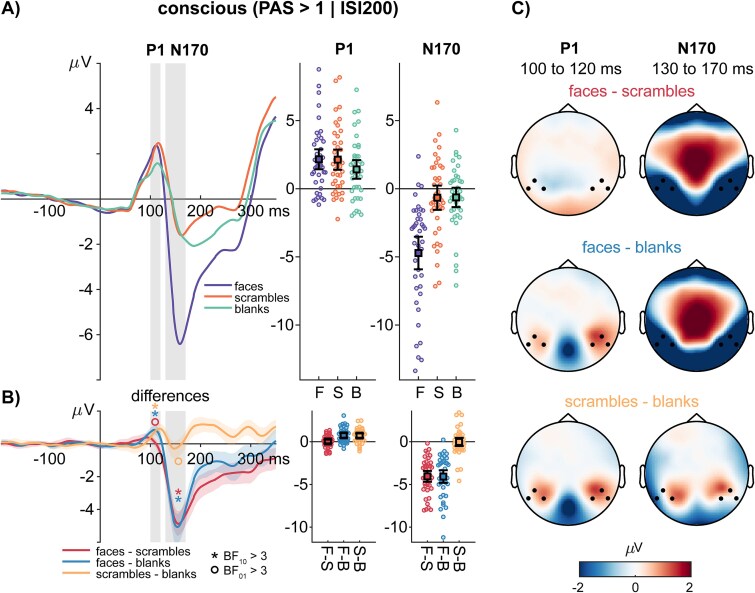
ERPs for conscious stimuli: (A) average ERPs to conscious faces (F), scrambles (S), and blanks (B). The gray vertical bars highlight the interval defined for calculating average P1 and N170 amplitudes, respectively. The error bar plots to the left of the ERPs show the average amplitudes and the 95% confidence interval of the mean. Colored dots represent average amplitudes of individual participants. (B) Difference wave forms for faces minus scrambles, faces minus blanks, and scrambles minus blanks. The shaded regions around the difference waves indicate the 95% bootstrap confidence interval of the average difference waves. The error bar plots to the left of the difference waves show the average amplitude differences and the 95% confidence interval of the mean difference between the respective conditions. The asterisk and circle symbol are used to highlight where the Bayes factor indicated at least moderate evidence in favor of the null (○: BF_01_ > 3) or alternative hypothesis (*: BF_01_ > 3). (C) Difference topographies showing the average amplitude differences in the selected intervals. Black dots highlight the channels of interest chosen for the ERP analysis.

**Figure 4:**
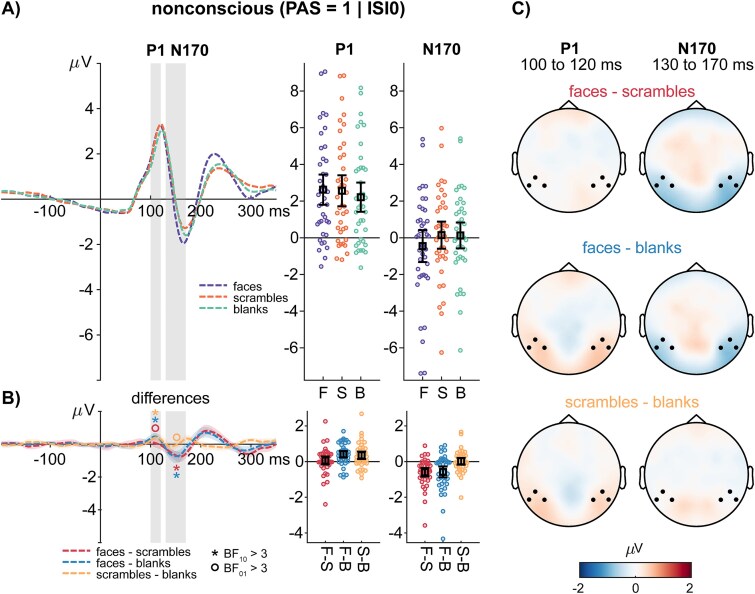
ERPs for nonconscious stimuli: (A) average ERPs to conscious faces, scrambles, and blanks. The gray vertical bars highlight the interval defined for calculating average P1 and N170 amplitudes, respectively. The error bar plots to the left of the ERPs show the average amplitudes and the 95% confidence interval of the mean. Colored dots represent average amplitudes of individual participants. (B) Difference wave forms for faces minus scrambles, faces minus blanks and scrambles minus blanks. The shaded regions around the difference waves indicate the 95% bootstrap confidence interval of the average difference waves. The error bar plots to the left of the difference waves show the average amplitude differences and the 95% confidence interval of the mean difference between the respective conditions. The asterisk and circle symbol are used to highlight where the Bayes factor indicated at least moderate evidence in favor of the null (○: BF_01_ > 3) or alternative hypothesis (*: BF_01_ > 3). (C) Difference topographies showing the average amplitude differences in the selected intervals. Black dots highlight the channels of interest chosen for the ERP analysis.

**P1:** For the P1, we observed a main effect of stimulus type (F_(2,78)_ = 25.336, *P* < .001, η_P_^2^ = .394). As shown in Fig. 3 and Fig. 4, average P1 amplitudes were descriptively similar for faces and scrambles (M_faces_ = 2.393, SD = 2.543; *M*_scrambles_ = 2.349, SD = 2.586) and lower for blanks (*M*_blanks_ = 1.817, SD = 2.444). A main effect of consciousness (F_(1,39)_ = 19.256, *P* < .001, η_P_^2^ = .331) revealed that P1 amplitudes were, on average, higher in the nonconscious (*M*_nonconscious_ = 2.467, SD = 2.670) compared to the conscious condition (*M*_conscious_ = 1.905, SD = 2.355). Finally, we observed an interaction of stimulus type and consciousness (F_(2,78)_ = 3.725, *P* = .029, η_P_^2^ = 0.087).

Among consciously perceived faces planned comparisons using pairwise Bayesian t-tests revealed extreme evidence for higher P1 amplitudes in response to faces compared to blanks (BF_10_ = 16500.353, error < 0.001%) and for higher P1 amplitudes to scrambles compared to blanks (BF_10_ = 18624.230, error < 0.001%). We also obtained moderate evidence for the absence of a P1 amplitude difference between faces and scrambles (BF_01_ = 5.695, error < 0.001%; see Fig. 3).

In the unconscious condition, we observed extreme evidence for higher P1 amplitudes to faces compared to blanks (BF_10_ = 376.941, error < 0.001%) and strong evidence for higher P1 amplitudes to scrambles compared to blanks (BF_10_ = 26.421, error < 0.001%). Finally, we obtained moderate evidence for the absence of a P1 amplitude difference between faces and scrambles (BF_01_ = 5.169, error < 0.001%; see Fig. 4).

To resolve the interaction effect of stimulus type and consciousness, we compared face-blank and scramble-blank differences between the conscious and nonconscious conditions. We observed moderate evidence for larger face-blank differences in the conscious compared to the nonconscious condition (BF_10_ = 3.377, error < 0.001%) and moderate evidence for larger scramble-blank differences in the conscious compared to the nonconscious condition (BF_10_ = 5.757, error < 0.001%).

**N170:** For the N170, we observed a main effect of stimulus type (F_(2,78)_ = 89.435, *P* < .001, η_P_^2^ = 0.696). Figures 3 and 4 indicate that N170 amplitudes were, on average, largest for faces (*M*_faces_ = −2.584, SD = 4.019), followed by scrambles (*M*_scrambles_ = −0.257, SD = 2.685) and then blanks (*M*_blanks_ = −0.254, SD = 2.339). There was a main effect of consciousness (*F*_(1,39)_ = 64.213, *P* < .001, η_P_^2^ = 0.622) due to higher N170 amplitudes in the conscious (*M*_conscious_ = −2.003, SD = 3.643) compared to the nonconscious condition (*M*_nonconscious_ = −0.061, SD = 2.529). Finally, there was a significant interaction of stimulus type and consciousness (*F*_(2,78)_ = 96.297, *P* < .001, η_P_^2^ = 0.712).

In the conscious condition, planned comparisons revealed extreme evidence for higher N170 amplitudes to faces compared to blanks (BF_10_ = 2.340 × 10^12^, error < 0.001%) and for higher amplitudes to faces compared to scrambles (BF_10_ = 3.753 × 10^10^, error < 0.001%). We also obtained moderate evidence for the absence of an N170 amplitude difference between scrambles and blanks (BF_01_ = 5.839, error < 0.001%; see Fig. 3).

In the unconscious condition, we observed extreme evidence for higher N170 amplitudes to faces compared to scrambles (BF_10_ = 1786.315, error < 0.001%) and strong evidence for higher amplitudes to faces compared to blanks (BF_10_ = 79.753, error < 0.001%). Finally, we obtained moderate evidence for the absence of an N170 amplitude difference between scrambles and blanks (BF_01_ = 5.773, error < 0.001%; see Fig. 4).

Again, to resolve the interaction effect of stimulus type and consciousness, we compared face-scramble and face-blank differences between the conscious and nonconscious conditions. We observed extreme evidence for larger face-scramble differences in the conscious compared to the nonconscious condition (BF_10_ = 8.071 × 10^11^, error < 0.001%) and extreme evidence for larger scramble-blank differences in the conscious compared to the nonconscious condition (BF_10_ = 5.845 × 10^10^, error < 0.001%).

## Discussion

Our study showed that the P1 and the N170 index different stages of face processing that were both observable when stimuli were rendered nonconscious by backward masking. While the P1 was associated with face-unspecific low-level differentiation between stimuli of different contrast modulation, the N170 was related to high-level configural face-processing. In both cases, backward masking strongly attenuated differential processing but was still reliably observed in trials in which participants indicated to have perceived nothing but the mask. Since nonconscious effects appear to be roughly two to five times smaller than the corresponding conscious effects, they may have been easily overlooked in lower-powered studies (*N* = 16; Retter et al. 2020; *N* = 11; Rodríguez et al. 2012).

These results replicate and extend our previous study (Bruchmann et al. 2025), in which we found clear evidence for face-blank differentiation in the N170 in the nonconscious condition. In this previous study, we also observed P1 differences between masked faces and blanks, albeit only in an exploratory analysis outside the preregistered interval of interest, calling for replication. With the additional comparison to low-level matched scrambles, we can now dissociate stimulus differentiation based on stimulus contrast and stimulus differentiation based on configural stimulus properties: the former process is indicated by P1 differences between blanks and both faces and scrambles and the absence of P1 differences between faces and scrambles. The latter process is indicated by N170 differences between faces and scrambles.

Our results are also largely in line with those reported by Harris et al. (2011), who compared faces, houses, and blanks in a sandwich-masking paradigm. The authors also found P1 differences between masked stimuli with and without contrast modulation (faces and houses vs. blanks) and N170 differences between masked faces and houses. As in our study, the differences between masked stimuli were strongly reduced compared to the corresponding effects in the unmasked condition. However, Harris et al. (2011) relied on the effectiveness of masking by testing face-house discrimination performance on the group level, which leaves open the possibility that some trials in which some participants became aware of the masked stimuli drove the residual effects observed in the masked condition. Our results, however, support the interpretation that residual effects remain even when analyzing only trials in which participants reported their subjective unawareness of the targets. Harris and colleagues attributed the attenuation of the effects by masking to the forward mask, which is assumed to interfere with the feedforward signal of the target (Breitmeyer and Öğmen 2006) and not the backward mask, which is proposed to interfere with feedback signals into early visual areas (Fahrenfort et al. 2007). Our results contradict this interpretation as we observed a comparable attenuation of P1 and N170 differences with only a backward mask. We conclude that it is more plausible to assume that the backward mask disrupts reentrant processing (Lamme 2003, 2006, 2018) to a degree that prevents the stimulus from being consciously perceived while leaving enough stimulus information intact to allow both low-level and some high-level processing.

Based on their timing, P1 and N170 effects can be attributed to different cortical regions in the face processing network (Duchaine and Yovel 2015). EEG amplitudes around 110 ms have been shown to correlate with the activity in the occipital face area (OFA), while face selectivity around 170 ms correlates with activity in the fusiform face area (FFA; Dellert et al. 2021) and the posterior superior temporal sulcus (pSTS; Sadeh et al. 2010). Within the face processing network, OFA has been proposed to represent view-specific and part-based information, pSTS to process facial expressions, and the FFA to represent holistic facial information (Duchaine and Yovel 2015). We conclude that backward masking strongly attenuates OFA, FFA, and pSTS activation, but leaves sufficient information for OFA to differentiate between stimuli with and without contrast modulation and for FFA to differentiate between faces and scrambles. Our previous study also showed N170 differences between unseen fearful and neutral faces (Bruchmann et al. 2025), and these expression effects may thus be attributable to the pSTS.

As outlined in the Introduction, the relationship between conscious perception and face-selective EEG signals has been studied using a variety of experimental approaches, revealing a heterogeneous pattern of results. In studies comparing stimuli differing in low-level properties, P1 effects appear to be observable in the absence of awareness (Harris et al. 2011; Bruchmann et al. 2025). Regarding N170 differences between faces and low-level-controlled stimuli, the picture is more complicated. Under conditions of inattentional blindness, N170 differences have not been found (Shafto and Pitts 2015; Dellert et al. 2021). In these cases, however, random line patterns were compared with patterns in which some of the lines formed the outline of facial features, creating a comparably subtle face stimulus. Correspondingly, the resulting N170 differences in the conscious condition were roughly two (Shafto and Pitts 2015) to four (Dellert et al. 2021) times smaller than in the present study. If nonconscious effects resemble fractions of the conscious effects, as we suggest here, they might be undetectably small in studies with weak stimuli. Furthermore, these inattentional blindness studies required participants to attend away from the stimuli, whereas faces were in the center of attention in the present study. Nonconscious effects may thus depend on attention (Axelrod et al. 2015).

CFS studies showed increased (Sterzer et al. 2009) as well as decreased (Suzuki and Noguchi 2013) responses to suppressed faces compared to non-faces in the N170 time range. Similar to the inattentional blindness studies, stimulus strength might be an important factor here as well, as suggested by a recent study from our group (Schlossmacher et al. 2025), which compared fearful and neutral faces under CFS and found that differences in the N170 range are modulated by the faces' brightness contrast.

A recent study compared ERPs to faces and objects and tested the limits of nonconscious perception using extremely brief (≤4.3 ms), unmasked stimulus presentations (Lanfranco et al. 2024). Using this design, the potential effects of masks and target-mask interactions in CFS or backward masking studies are avoided. On the other hand, stimulus strength varies with conscious detection in designs, which use no interfering attentional manipulation or stimulus. The authors observed no P1 differences, in line with their approach to match faces and objects in brightness and contrast. They observed N170 differences but only for stimulus durations > 2.45 ms. With 2.45 ms, however, participants were already subjectively aware of the stimuli and could objectively discriminate faces from objects above chance level (see Supplementary Fig. 23 in Lanfranco et al. 2024). These results implicate that a minimal exposure duration is necessary to observe face-specific N170 effects. It remains open whether these effects completely disappear in subjectively conscious vs. unconscious trials for a given stimulus duration. Future studies might directly compare findings from different designs, varying both signal strength and attentional or perceptual interference, to better understand the varying thresholds for nonconscious effects and measures of detection performance.

Taken together, the findings suggest that sufficient study power and a suitable operationalization of awareness might improve research on nonconscious stimulus processing. Furthermore, nonconscious effects seem to be much weaker than effects for conscious stimuli, but to reflect similar stages of processing depending on low (P1) vs. high level (N170) features.

We would like to note some limitations of our study. First, the behavioral data show that the faces were, on average, detectable above chance level in the ISI0 condition, confirming previous findings we obtained with a similar paradigm (Bruchmann et al. 2025). Although this difference may also stem from nonconscious processes (Merikle et al. 2001), it may also indicate residual awareness of the stimuli. By obtaining trial-by-trial PAS ratings, we sought to include only those from the ISI0 condition in which participants were subjectively unaware of the faces. However, despite clearly defined PAS labels, participants may still differ in their individual response criteria. Although the exclusion of participants with extreme biases limits this variability, we cannot fully exclude that the nonconscious conditions contain some proportion of weakly conscious trials from participants with conservative response criteria. As recently argued by Michel (2023), however, assigning PAS = 2-trials to the nonconscious condition could also lead to an underestimation of nonconscious processing, as the task-relevant feature, i.e. a face, is not consciously perceived even in these trials. Second, we only investigate one specific “blinding” paradigm. The extent of nonconscious processing may depend critically on the way conscious perception is manipulated, and other paradigms such as the attentional blink, or inattentional blindness may allow for stronger nonconscious processing than backward masking (Axelrod et al. 2015; Breitmeyer 2015). Furthermore, we did not compare faces to other high level categories. By doing so systematically, future studies could provide a more detailed understanding of the interplay of consciousness and face processing.

## Conclusion

This study showed that both conscious and nonconscious face processing modulate the P1 and the N170. We observed low-level stimulus differentiation at the level of the P1 and configural face processing at the level of the N170. In both cases, the effects observed in the nonconscious condition are temporally and topographically highly similar to those observed in the conscious condition but are considerably smaller. These results suggest that evidence for electrophysiological correlates of nonconscious face processing requires high-powered studies and should use strong stimuli, which should optimally produce large effects when consciously perceived.

## Supplementary Material

OPEN_SCIENCE_BADGE_APPLICATION_FORM_niaf025

Supplement_niaf025

## Data Availability

Raw data and pre-processed ERPs are available at https://osf.io/tajn9/

## References

[ref1] Axelrod V, Bar M, Rees G. Exploring the unconscious using faces. Trends Cogn Sci 2015;19:35–45. 10.1016/j.tics.2014.11.003 25481216

[ref2] Benjamini Y, Hochberg Y. Controlling the false discovery rate: a practical and powerful approach to multiple testing. J R Stat Soc Ser B Methodol 1995;57:289–300. 10.1111/j.2517-6161.1995.tb02031.x

[ref3] Bentin S, Allison T, Puce A et al. Electrophysiological studies of face perception in humans. J Cogn Neurosci 1996;8:551–65. 10.1162/jocn.1996.8.6.551 20740065 PMC2927138

[ref4] Brainard DH . The psychophysics toolbox. Spat Vis 1997;10:433–6. 10.1163/156856897X00357 9176952

[ref5] Breitmeyer BG . Psychophysical “blinding” methods reveal a functional hierarchy of unconscious visual processing. Conscious Cogn 2015;35:234–50. 10.1016/j.concog.2015.01.012 25704454

[ref6] Breitmeyer BG, Öğmen H. Visual Masking: Time Slices through Conscious and Unconscious Vision. Oxford: Oxford University Press, 2006. 10.1093/acprof:oso/9780198530671.001.0001

[ref7] Bruchmann M, Schindler S, Straube T. The spatial frequency spectrum of fearful faces modulates early and mid-latency ERPs but not the N170. Psychophysiology. 2020;57:e13597. 10.1111/psyp.13597 32390215

[ref8] Bruchmann M, Schindler S, Breitwieser P et al. Early neural responses to nonconscious fearful and neutral faces – an ERP study (p. 2025.01.30.635452) bioRxiv. 2025. 10.1101/2025.01.30.635452

[ref8a] Cohen J . Statistical Power Analysis for the Behavioral Sciences. Routledge, 2013. 10.4324/9780203771587

[ref9] Civile C, Elchlepp H, McLaren R et al. The effect of scrambling upright and inverted faces on the N170. Q J Exp Psychol 2018;71:2464–76. 10.1177/1747021817744455 30362407

[ref10] Crouzet SM, Kirchner H, Thorpe SJ. Fast saccades toward faces: face detection in just 100 ms. J Vis 2010;10:1–17. 10.1167/10.4.16 20465335

[ref11] Dellert T, Müller-Bardorff M, Schlossmacher I et al. Dissociating the neural correlates of consciousness and task relevance in face perception using simultaneous EEG-fMRI. J Neurosci 2021;41:7864–75. 10.1523/JNEUROSCI.2799-20.2021 34301829 PMC8445054

[ref12] Dering B, Martin CD, Moro S et al. Face-sensitive processes one hundred milliseconds after picture onset. Front Hum Neurosci 2011;5:1–14. 10.3389/fnhum.2011.00093 PMC3173839 21954382

[ref13] Duchaine B, Yovel G. A revised neural framework for face processing. Annual Review of Vision Science 2015;1:393–416. 10.1146/annurev-vision-082114-035518 28532371

[ref14] Ebner NC, Riediger M, Lindenberger U. FACES--a database of facial expressions in young, middle-aged, and older women and men: development and validation. Behav Res Methods 2010;42:351–62. 10.3758/BRM.42.1.351 20160315

[ref15] Eimer M . The face-specific N170 component reflects late stages in the structural encoding of faces. Neuroreport 2000;11:2319–24. 10.1097/00001756-200007140-00050 10923693

[ref16] Fahrenfort JJ, Scholte HS, Lamme VAF. Masking disrupts reentrant processing in human visual cortex. J Cogn Neurosci 2007;19:1488–97. 10.1162/jocn.2007.19.9.1488 17714010

[ref17] Fahrenfort JJ, Johnson PA, Kloosterman NA et al. Criterion placement threatens the construct validity of neural measures of consciousness. eLife 2025;13:RP102335. 10.7554/eLife.102335.4 40434818 PMC12119085

[ref18] Fleming SM . HMeta-d: hierarchical Bayesian estimation of metacognitive efficiency from confidence ratings. Neurosci Conscious 2017;2017:1–14. 10.1093/nc/nix007 PMC5858026 29877507

[ref19] Green DM, Swets JA. Signal Detection Theory and Psychophysics. New York: Wiley, 1966.

[ref20] Harris JA, Wu C-T, Woldorff MG. Sandwich masking eliminates both visual awareness of faces and face-specific brain activity through a feedforward mechanism. J Vis 2011;11:3. 10.1167/11.7.3 PMC3513353 21669859

[ref21] Hautus MJ, Macmillan NA, Creelman CD. Detection Theory: A User’s Guide3rd edn. New York: Routledge, 2021. 10.4324/9781003203636

[ref22] Ille N, Berg P, Scherg M. Artifact correction of the ongoing EEG using spatial filters based on Artifact and brain signal topographies. J Clin Neurophysiol 2002;19:113–24. 10.1097/00004691-200203000-00002 11997722

[ref23] Itier RJ, Taylor MJ. N170 or N1? Spatiotemporal differences between object and face processing using ERPs. Cereb Cortex 2004;14:132–42. 10.1093/cercor/bhg111 14704210

[ref24] Jiang Y, Costello P, He S. Processing of invisible stimuli: advantage of upright faces and recognizable words in overcoming Interocular suppression. Psychol Sci 2007;18:349–55. 10.1111/j.1467-9280.2007.01902.x 17470261

[ref25] Jiang Y, Shannon RW, Vizueta N et al. Dynamics of processing invisible faces in the brain: automatic neural encoding of facial expression information. Neuroimage 2009;44:1171–7. 10.1016/j.neuroimage.2008.09.038 18976712 PMC3180886

[ref26] Kerr JA, Hesselmann G, Räling R et al. Choice of analysis pathway dramatically affects statistical outcomes in breaking continuous flash suppression. Sci Rep 2017;7:3002. 10.1038/s41598-017-03396-3 28592830 PMC5462748

[ref27] Keysers C, Gazzola V, Wagenmakers E-J. Using Bayes factor hypothesis testing in neuroscience to establish evidence of absence. Nat Neurosci 2020;23:788–99. 10.1038/s41593-020-0660-4 32601411 PMC7610527

[ref28] Kleiner M, Brainard DH, Pelli DG. What’s new in Psychtoolbox-3? Perception 2007;36:14.

[ref29] Lamme VAF . Why visual attention and awareness are different. Trends Cogn Sci 2003;7:12–8. 10.1016/S1364-6613(02)00013-X 12517353

[ref30] Lamme VAF . Towards a true neural stance on consciousness. Trends Cogn Sci 2006;10:494–501. 10.1016/j.tics.2006.09.001 16997611

[ref31] Lamme VAF . Challenges for theories of consciousness: seeing or knowing, the missing ingredient and how to deal with panpsychism. Philos Trans R Soc B 2018;373:20170344. 10.1098/rstb.2017.0344 PMC6074090 30061458

[ref32] Lanfranco RC, Rabagliati H, Carmel D. The importance of awareness in face processing: a critical review of interocular suppression studies. Behav Brain Res 2023;437:114116. 10.1016/j.bbr.2022.114116 36113728

[ref33] Lanfranco RC, Canales-Johnson A, Rabagliati H et al. Minimal exposure durations reveal visual processing priorities for different stimulus attributes. Nat Commun 2024;15:8523. 10.1038/s41467-024-52778-5 39358365 PMC11447214

[ref34] Langner O, Dotsch R, Bijlstra G et al. Presentation and validation of the Radboud faces database. Cognit Emot 2010;24:1377–88. 10.1080/02699930903485076

[ref35] Lee MD, Wagenmakers E-J. Bayesian Cognitive Modeling: A Practical Course(1st ed.). Cambridge: Cambridge University Press, 2014. 10.1017/CBO9781139087759

[ref36] Macmillan NA, Kaplan HL. Detection theory analysis of group data: estimating sensitivity from average hit and false-alarm rates. Psychol Bull 1985;98:185–99. 10.1037/0033-2909.98.1.185 4034817

[ref37] Maniscalco B, Lau H. A signal detection theoretic approach for estimating metacognitive sensitivity from confidence ratings. Conscious Cogn 2012;21:422–30. 10.1016/j.concog.2011.09.021 22071269

[ref38] Maniscalco B, Lau H. Signal detection theory analysis of type 1 and type 2 data: Meta-d′, response-specific meta-d′, and the unequal variance SDT model. In: Fleming SM, Frith CD (eds.), The Cognitive Neuroscience of Metacognition, pp. 25–66. Berlin Heidelberg: Springer, 2014 10.1007/978-3-642-45190-4_3

[ref39] Merikle PM, Smilek D, Eastwood JD. Perception without awareness: perspectives from cognitive psychology. Cognition 2001;79:115–34. 10.1016/S0010-0277(00)00126-8 11164025

[ref40] Michel M . How (not) to underestimate unconscious perception. Mind Lang 2023;38:413–30. 10.1111/mila.12406

[ref41] Moors, P. What’s up with high-level processing during continuous flash suppression? In: Hesselmann G (ed.), Transitions between Consciousness and Unconsciousness*,* 1st edn. New York: Routledge/Taylor & Francis Group, 2019, 39–70. 10.4324/9780429469688-2

[ref42] Moors P, Gayet S, Hedger N et al. Three criteria for evaluating high-level processing in continuous flash suppression. Trends Cogn Sci 2019;23:267–9. 10.1016/j.tics.2019.01.008 30795895

[ref43] Mudrik L, Deouell LY. Neuroscientific evidence for processing without awareness. Annu Rev Neurosci 2022;45:403–23. 10.1146/annurev-neuro-110920-033151 35803585

[ref44] Overgaard M, Sandberg K. The perceptual awareness scale—recent controversies and debates. Neurosci Conscious 2021;2021:niab044. 10.1093/nc/niab044 34925909 PMC8672240

[ref45] Overgaard M, Timmermans B, Sandberg K et al. Optimizing subjective measures of consciousness. Conscious Cogn 2010;19:682–4. 10.1016/j.concog.2009.12.018 20097582

[ref46] Prieto A, Montoro PR, Jimenez M, Hinojosa JA. In Search of an Integrative Method to Study Unconscious Processing: An Application of Bayesian and General Recognition Theory Models to the Processing of Hierarchical Patterns in the Absence of Awareness. Journal of Cognition 2025;8:1–34. 10.5334/joc.411 39803183 PMC11720486

[ref47] Retter TL, Jiang F, Webster MA et al. All-or-none face categorization in the human brain. Neuroimage 2020;213:116685. 10.1016/j.neuroimage.2020.116685 32119982 PMC7339021

[ref48] Rodríguez V, Thompson R, Stokes M et al. Absence of face-specific cortical activity in the complete absence of awareness: converging evidence from functional magnetic resonance imaging and event-related potentials. J Cogn Neurosci 2012;24:396–415. 10.1162/jocn_a_00137 21942763

[ref49] Rossion B, Caharel S. ERP evidence for the speed of face categorization in the human brain: disentangling the contribution of low-level visual cues from face perception. Vis Res 2011;51:1297–311. 10.1016/j.visres.2011.04.003 21549144

[ref50] Rousselet GA, Macé MJ-M, Fabre-Thorpe M. Spatiotemporal analyses of the N170 for human faces, animal faces and objects in natural scenes. Neuroreport 2004;15:2607–11. 10.1097/00001756-200412030-00009 15570161

[ref51] Sadeh B, Podlipsky I, Zhdanov A et al. Event-related potential and functional MRI measures of face-selectivity are highly correlated: a simultaneous ERP-fMRI investigation. Hum Brain Mapp 2010;31:1490–501. 10.1002/hbm.20952 20127870 PMC6870976

[ref52] Sandberg K, Overgaard M. Using the perceptual awareness scale (PAS). In: Overgaard M (ed.), Behavioral Methods in Consciousness Research, pp. 181–96. Oxford: Oxford University Press, 2015. 10.1093/acprof:oso/9780199688890.003.0011

[ref53] Schindler S, Bruchmann M, Gathmann B et al. Effects of low-level visual information and perceptual load on P1 and N170 responses to emotional expressions. Cortex 2021;136:14–27. 10.1016/j.cortex.2020.12.011 33450599

[ref54] Schlossmacher I, Junghöfer M, Straube T et al. No differential effects to facial expressions under continuous flash suppression: an event-related potentials study. Neuroimage 2017;163:276–85. 10.1016/j.neuroimage.2017.09.034 28939431

[ref55] Schlossmacher I, Herbig M, Dellert T et al. The influence of signal strength on conscious and nonconscious neural processing of emotional faces. Neurosci Conscious 2025;2025:niaf001. 10.1093/nc/niaf001 39916700 PMC11799861

[ref56] Shafto JP, Pitts MA. Neural signatures of conscious face perception in an Inattentional blindness paradigm. J Neurosci 2015;35:10940–8. 10.1523/JNEUROSCI.0145-15.2015 26245958 PMC6605277

[ref57] Stein T, Peelen MV. Dissociating conscious and unconscious influences on visual detection effects. Nat Hum Behav 2021;5:612–24. 10.1038/s41562-020-01004-5 33398144

[ref58] Sterzer P, Jalkanen L, Rees G. Electromagnetic responses to invisible face stimuli during binocular suppression. Neuroimage 2009;46:803–8. 10.1016/j.neuroimage.2009.02.046 19285140

[ref59] Sterzer P, Stein T, Ludwig K et al. Neural processing of visual information under interocular suppression: a critical review. Front Psychol 2014;5:1–12. 10.3389/fpsyg.2014.00453 PMC4032950 24904469

[ref60] Suzuki M, Noguchi Y. Reversal of the face-inversion effect in N170 under unconscious visual processing. Neuropsychologia 2013;51:400–9. 10.1016/j.neuropsychologia.2012.11.021 23196145

[ref61] JASP Team . *JASP* (Version 0.7.5.5) [Computer Software] 2016.

[ref62] Thierry G, Martin CD, Downing P et al. Controlling for interstimulus perceptual variance abolishes N170 face selectivity. Nat Neurosci 2007;10:505–11. 10.1038/nn1864 17334361

[ref63] Tottenham N, Tanaka JW, Leon AC et al. The NimStim set of facial expressions: judgments from untrained research participants. Psychiatry Res 2009;168:242–9. 10.1016/j.psychres.2008.05.006 19564050 PMC3474329

[ref64] Wang H, Sun P, Ip C et al. Configural and featural face processing are differently modulated by attentional resources at early stages: an event-related potential study with rapid serial visual presentation. Brain Res 2015;1602:75–84. 10.1016/j.brainres.2015.01.017 25601005

[ref65] Yang E, Zald DH, Blake R. Fearful expressions gain preferential access to awareness during continuous flash suppression. Emotion 2007;7:882–6. 10.1037/1528-3542.7.4.882 18039058 PMC4038625

