# Supplementary material for: Early ERPs dissociate subjectively nonconscious low- and high-level face processing
Source: Neurosci Conscious. 2025 Aug 19;2025(1):niaf025. doi: 10.1093/nc/niaf025 (PMC12363218; doi:10.1093/nc/niaf025)
Supplement: OPEN_SCIENCE_BADGE_APPLICATION_FORM_niaf025 [file open_science_badge_application_form_niaf025.docx]

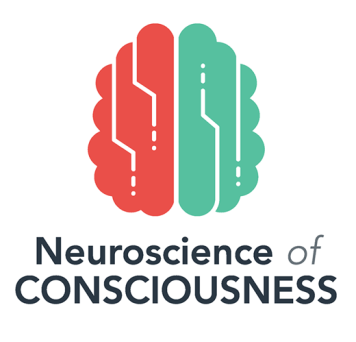
 **OPEN SCIENCE BADGE APPLICATION FORM**

**Open Data Badge**

Please provide the URL, DOI, or other permanent path for accessing the data in a public, open access repository.

I

https://osf.io/tajn9/

Is there sufficient information for an independent researcher to reproduce the reported results? If no, explain.

yes

**Open Materials Badge**

Please provide the URL, DOI, or other permanent path for accessing the materials in a public, open access repository.

I

https://osf.io/tajn9/

Is there sufficient information for an independent researcher to reproduce the reported methodology? If no, explain.

No. Decisions regarding which electrodes to interpolate were based on eye inspection of experienced researchers, as was the individually chosen threshold for artefacts due to high amplitudes.

**Preregistered Badge**

Please provide the URL, DOI, or other permanent path to the registration (and, if applicable, the analysis plan) in a public, open access repository.

I

https://osf.io/tajn9/

Was the plan preregistered prior to the examination of the data or observing the outcomes? If no, explain.

yes

Were there additional registrations for the study other than the one reported? If yes, provide links and explain.

no

For Preregistered and Analysis plan badge: were there any changes to the preregistered analysis plan for the primary confirmatory analysis? If yes, explain.

No

For Preregistered and Analysis plan badge: are all of the analyses described in the registered plan reported in the article? If no, explain.

Yes
