# Supplementary material for: Early ERPs dissociate subjectively nonconscious low- and high-level face processing
Source: Neurosci Conscious. 2025 Aug 19;2025(1):niaf025. doi: 10.1093/nc/niaf025 (PMC12363218; doi:10.1093/nc/niaf025)
Supplement: Supplement_niaf025 [file supplement_niaf025.pdf]

Supplementary material

Early ERPs dissociate subjectively nonconscious low- and high-level face processing

Maximilian Bruchmann[1,2†*], Josephine Skutta[1†], Sebastian Schindler[1,2], Insa Schlossmacher[1,2], Torge Dellert[1,2], and Thomas Straube[1]

Generation of spectrum-matched phase-scrambles faces

Phase-scrambling an image is achieved by applying Fourier analysis to an image, producing the image's power and phase spectrum. The phase spectrum is then typically replaced by that of a random noise image. The original power spectrum and the random phase spectrum are then recombined by the inverse Fourier transform or Fourier synthesis. Since Fourier analysis and synthesis operate on rectangular image matrices, classical phase-scrambling produces rectangular scrambles, even if the input image contains an oval-cutout phase. The power spectrum of the rectangular scramble is then identical to the power spectrum of the rectangular input image. Applying the same oval-cutout mask to the scramble would preserve the shape of the face in the input image but would remove image parts and thus reduce the power of the spatial frequencies contained in these regions. The result would be an image with an oval-cutout scramble whose spectrum is no longer identical to the input image's spectrum. To produce scrambles with the same oval shape as the faces but with an optimally matched power spectrum with the oval target region, we applied the following algorithm to each face:

1. The image was cut to its minimal rectangular size, removing all pixel rows and columns that consist only of the background color. This reduced the amount of spectral power contributed by the background.
2. A fast Fourier transform (Matlab function fft2) yielded the power and phase spectrum of the minimized image.

3. A noise matrix with the same image size as the minimized image was created (Matlab function rand) consisting of uniformly distributed random values between 0 and 1 per pixel.
4. A fast Fourier transform yielded the power and phase spectrum of the noise matrix.
5. A rectangular scrambled image was created by inverse Fourier transformation (ifft2) using the minimized image's power spectrum and the noise image's phase spectrum.
6. The oval cutout with blurred edges was applied to the rectangular scramble. Note that this removes about 30% of the image and thus its spectral power. Furthermore, since the distribution of frequencies across the rectangular image was randomized, the removed regions may have contained potentially relevant frequency information.
7. The amplitude spectrum of the resulting oval scramble was calculated (fft2) and compared with the power spectrum of the original oval face by calculating the sum of squares of the differences between these spectra as a measure of dissimilarity between the power spectra.
8. Steps 3 to 7 were repeated 1.000.000 times, and the iteration with the highest similarity (lowest SSQ) was chosen as the winning phase scramble.
9. The contrast of the winning scramble was increased in steps of 0.1% from 100% to 200%, each time calculating the same dissimilarity metric described above to find the contrast level at which the scramble's power spectrum optimally matched the corresponding face's power spectrum.
10. The optimized oval scramble was saved as an image file, yielding one optimally matching scramble per face.
